# Who counts when health counts? A case-study of multi-stakeholder initiative to promote value-creation in Swedish healthcare

**DOI:** 10.1177/09514848221100751

**Published:** 2022-05-16

**Authors:** Leonard Tragl, Carl Savage, Magna Andreen-Sachs, Mats Brommels

**Affiliations:** Medical Management Centre, Department of Learning, Informatics, Management and Ethics (LIME), 27106 Karolinska Institute, Sweden

**Keywords:** multi-stakeholder, innovations, collaboration, networks, health care, value-based healthcare

## Abstract

A European initiative to design a “medical information framework” conceptualised how multiple stakeholders join in collaborative networks to create innovations. It conveyed the ways in which value is created and captured by stakeholders. We applied those insights to analyse a multi-stakeholder initiative to promote improvement of Swedish healthcare. Our longitudinal case study covered totally fifty stakeholders involved in a national project, aiming at designing a system to support value-based evaluation and reimbursement. During the project the focus changed from reimbursement to benchmarking. Sophisticated case-mix adjusting algorithms were designed to make outcome comparisons valid and incorporated in a software platform enabling detailed analysis of eight patient groups across seven regional health authorities. Those were deliverables demonstrating value created. However, the project was unable to transfer the system into routine use in the regions, a failed value-capture. The initial success was promoted by collaborative processes in diagnosis-specific working groups of well-informed and engaged professionals. The change of focus away from reimbursement decreased the involvement among health authorities, leaving no centrally placed persons to push for implementation. It highlights the importance of health professionals as the key stakeholder, who has both the know-how instrumental to creating an innovation, and the local involvement guaranteeing its implementation.

## Introduction

The publication of Porter’s and Teisberg’s book “Redefining Health Care: Creating Value-Based Competition on Results”^1^ led to wide-spread interest in outcomes measurement and “value-based” forms of healthcare management. Although the implementation of “value-based health care” (VBHC) often was based on shallow knowledge of concepts and methods,^2^ it did not limit the enthusiasm for an approach having indisputable face value: to reward service providers for clinical effectiveness rather than financial targets. Hurst et al.^3^ define VBHC as “an equitable, sustained, transparent use of available resources to achieve better outcomes and experience for every patient”. The authors identify key challenges and barriers to reach that ideal state. It requires better data than presently are available, better evidence on what works, an organised process for organisational change, multidisciplinary engagement, a unifying culture around value and governance systems focusing on delivering value over controlling costs and performance targets.^3^ That culture and system also require a clearer patient focus: simply measuring clinically defined measures of effectiveness and cost-effectiveness should be replaced with outcomes that patients most value.^4^

Value-based health care has also caught the attention of political decision-makers. In the US the Affordable Care Act was said to “catalyse” VBHC – and apart from the central parts of the legislation – there was bipartisan support for the introduction of innovative value-based care models. One of those was the Bundled Payments for Care Improvement programme in orthopaedic surgery.^5^ Those authors stated that “the pursuit of value in health care is now front and center”.

The ideas were rapidly picked up in Sweden, where an established system of over 100 quality registries provides outcome data across a wide variety of diagnoses and procedures.^6^ In 2012, the Swedish Department of Health initiated a research and development project to improve existing and develop new systems for evaluating and financing healthcare services. In 2013, 7 (out of 21) regional health authorities joined the initiative together with researchers at Karolinska Institutet and a private research-based information technology company (Ivbar Institute AB), and the SVEUS R&D project was formalised. Several Swedish national quality registries,^7^ professional organisations, patient organisations and two additional government agencies decided to participate.

This initiative was well placed to take on the challenges outlined by Hurst et al.,^3^ thanks to its involvement of multiple stakeholders and multidisciplinary spectrum of competences. Its bold ambition could well be defined, by using the language of Rühli et al.^8^ as “innovating around a complex socio-economic issue”, which calls for “broad stakeholder inclusion”. The emerging collaboration formed a “multi-stakeholder innovation network” based on voluntary collaboration.^9^

Much of collaboration in healthcare does take place in networks. Networks tend to be successful when stakeholders share a common vision, similar strategies and a history of having performed effectively.^10^ An additional perspective is offered by Sorensen et al.^11^ who promote value assessment frameworks, endorsed by multiple stakeholders involved, in order to enhance the *implementation* of innovations. Those frameworks, however, do not shed light on how stakeholders in collaboration will *develop* innovations in a shared ambition to create value.

## Aim

The aim of this study was to increase our understanding of how multiple stakeholders can collaboratively develop tools and processes to improve healthcare guided by value measurements.

## Theoretical framework

Guidance for the study was sought in an in-depth analysis of the European Medical Information Framework (EMIF) project that involved 57 public and private partners across 14 countries and engaging over 300 individuals.^9^ Its objective was to design an information framework that would enable access to patient-data “on a larger scale and at a higher level of detail than currently possible”. In this respect, EMIF resembles the SVEUS project, although SVEUS moved beyond frameworks to actual collection, integration, and analysis of clinical data from numerous sources. Although less complex in terms of its realm of work, EMIF engaged a slightly larger number of stakeholders in multiple countries.

In that project Reypens et al. explored how value is created in a *stakeholder network* and how stakeholders capture their share of the value.^9^ They identified three types of value outcomes. *Innovation outcomes* were new responses to problems to be solved in the network, in their case the information framework model and biomarkers in clinical application areas, which all were tangible deliverables. *Knowledge outcomes* were co-created by stakeholders, e.g. how to use technical tools, how to better understand the shared challenges and how to best organise the collaboration. *Relational outcomes* were emergent new connections between stakeholders. *Value co-creation* processes evolved on the network level as stakeholders interacted, contributed resources, and shared knowledge and experience. *Value capture* took place at the stakeholder level. It depended on the *anticipation* of the types of value to be created in order to find the right focus. Stakeholders assessed the value of the outcomes delivered in the collaboration as a basis for the final selection, which was followed by a process of transfer. Finally, the authors identified *boundary conditions* for a combined value leveraging to take place. The number of and differentiating characteristics of stakeholders made coordination challenging. Different cultures led to a diversity in language and connotations, making communication more difficult. Objectives and interests varied, from commercial to scientific. These differences also influenced their willingness to compromise.

**Figure 1.**
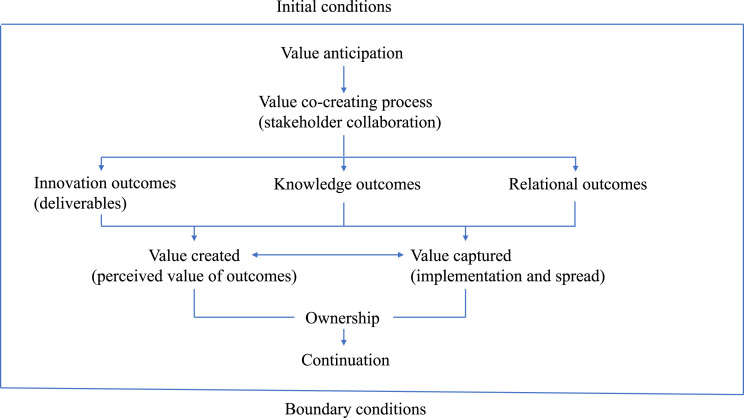
Schematic framework of stakeholder network.

We have brought together these concepts, their interrelations and the collaboration process of a stakeholder network in a framework, presented in Figure 1.

## Methodology

The research group was invited to monitor the SVEUS project throughout its 5-year lifespan. Consequently, the group had access to steering and project group meetings, all project documentation, and established contacts with representatives of all key stakeholders, and was thus in a good position to carry out an in-depth longitudinal case study of the project with an explanatory approach. That design was found suitable, as it is “an empirical inquiry that investigates a contemporary phenomenon within its real-life context; when the boundaries between phenomenon and context are not clearly evident; and in which multiple sources of evidence are used”.^12^

Data collection and analysis were guided by the multi-stakeholder innovation network model we derived from insights provided by Reypens et al. (Figure 1).^9^

The main advantage of applying this framework is that the study is grounded in a comprehensive conceptual model of multi-stakeholder innovation networks, thus enabling analytical generalisation of the findings of this single case. This should outweigh the limitations of directed data analysis, as additional viewpoints are possible to identify and further elaborate on when studying documents and performing interviews.

## Data collection

Data sources were participatory observations of steering group meetings, documents and interviews with stakeholder representatives who had given their verbal informed consent.

The main document was a project report in Swedish summarising the project and its results.^13^ It included a survey with 104 participants in the steering, expert, and working groups exploring views on project goals, outcomes, work process, and quality of the working group reports. Other documents were the benchmarking reports produced by the eight diagnosis groups.

12 persons, covering all stakeholder groups, were strategically selected for interviews. Selection was guided by our multiple stakeholder innovation network model (Figure 1). Interviewees represented the Department of Health, regional health authorities and quality registries, project and clinical sub-project leadership and expert groups.

## Data analysis

The information gathered by observations and documents were compiled into a chronological description of the project, highlighting critical events and reporting project outputs.

The initial directed content analysis on the verbatim interview transcripts was done by two of the authors not involved in the project.^14^ Transcripts were coded independently, but consensus reached on which meaning units to include and under which headings (themes) of the model. The development of categories and subcategories was performed by all authors, but without the ability for them to link meaning units to interviewees. Data were cross-checked, when needed, with the documents.

Mechanisms that described and explained “what worked, for whom and under which circumstances” were sought in the analysis and guided by the documents and the understanding researchers had gained during the project.^15^

The research plan, interview guide, and detailed written information to interviewees, was vetted by the Stockholm Regional Research Ethics Board (DNr 2019-05871).

## Findings

The results of the analysis is reported in two sections. First, the initiation, organising, execution and outputs of the SVEUS project are described. Second, the views of the interviewed stakeholders on their experience and perceived value of the project are presented, structured according to our theoretical framework based on insights provided by Reypens et al.^9^

### Case description

The SVEUS multi-stakeholder project ran from 2013 to 2017. It was, from its inception, designed as an R&D project after proper research ethics vetting and approval from relevant authorities, as the intention was to link databases. The two overarching research questions were:

1. How should future systems for evaluation and reimbursement of health services be designed in order to promote better health outcomes, and more efficient resource use, and reduce unwarranted variation?
2. How should these systems be routinely managed in Swedish healthcare?

An initial analysis, primarily based on previous experiences and access to data, resulted in the selection of eight patient groups: hip and knee replacement surgery, spine surgery, obesity surgery, obstetric care, diabetes, osteoporosis, and breast cancer. Seven regional health authorities, the Department of Health, the Swedish Association of Local Authorities and Regions, the State Social Insurance Institution, Statistics Sweden, Karolinska Institutet, 12 national quality registries, seven patient organisations and 19 medical, nursing and paramedical professional organisations participated in the project. Eight expert groups were established for each of the patient groups. Separate working groups were formed to analyse collaboration with Statistics Sweden and the Social Insurance Institution, which contributed data on social benefits, healthcare service utilisation, and living conditions. Over 150 single individuals were engaged in expert and working groups. The Ivbar Institute AB company – with competence in data management and sophisticated statistical analysis – was hired to manage the project.

Over time, the initial focus changed when regional health authorities found the introduction of “value-based” (health outcomes-related) reimbursement systems too difficult to achieve given the change in political climate. As a result, work was re-directed to develop systems for monitoring, assessing, and bench-marking performance data, to design management systems to that end, and to introduce those in the regional health authorities. Case-mix standardised performance indicators were designed, and benchmarking data were used for comparisons across regions and facilities for each patient group by teams involving patients, clinicians, registry representatives, and researchers. The company supported the expert groups and was responsible for data acquisition, management, analysis, and compiling summary reports. In addition, the company developed an “analysis platform” for use by the regional health authorities to further utilise the benchmarking information for improvement purposes.

The deliverables of the project were the analysis platform, applying algorithms derived for case-mix adjustment of outcomes in the eight patient groups, and comparisons of outcomes and resource use in the patient groups across provider organisations in the seven regions. Benchmarking studies were published in reports for all patient groups except obesity surgery. In addition, procedures describing the retrieval and use of Social Insurance Agency and Statistics Sweden data were documented and a plan for the integration of the analysis platform into regional patient administration systems was designed. 12 scientific studies and two PhD theses were also published.

As the project approached its conclusion, it sought to hand-over the analysis platform to enable regional health authorities to carry on with regular data collection, compilation, analysis, and reporting in order to utilise the project deliverables for continued benchmarking between regions and across clinical specialties. One of the participating regional health authorities took over management of the platform, and another region actively used the analysis tool as a support for process improvement of clinical services. In the end, the Swedish Association of Local Authorities and Regions (SALAR) declined to integrate the platform into its systems; without that support regions stopped using the service.

As the project ended, all 104 active participants were surveyed (response rate 45%). A majority of the respondents found that the research questions had been successfully addressed (65%) and the targets reached (55%), especially those on benchmarking (70%), although to a lesser extent the development of outcomes-based reimbursement systems (44%). The project organisation was rated as efficient or highly efficient by 70% of the participants, and participants were said to be highly competent and represent a good mix of expertise.^13^

The project reached all its stated goals and the deliverables were valued by the participants. The benchmarking reports stimulated improvement activities in all regions. In that respect, the project was successful. It failed, though, to establish the benchmarking system and its analysis platform and facilitate its adoption in routine use in the participating regions.

**Figure 2.**
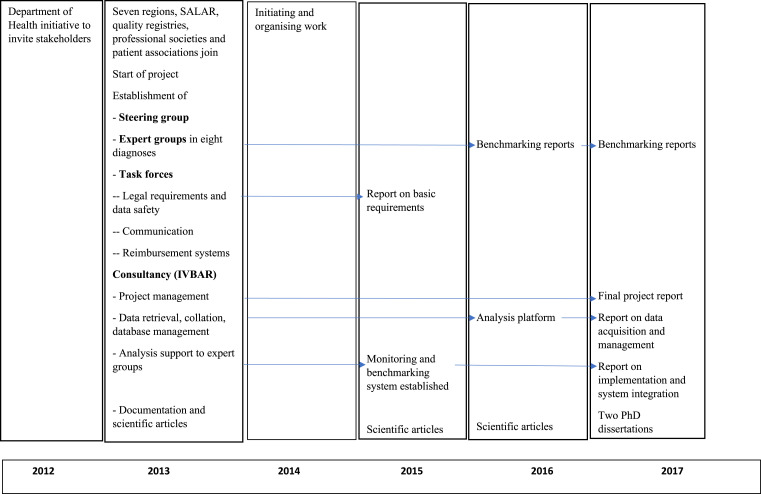
Timeline of key project events.

A timeline exhibit in Figure 2 summarises key events of the project.

### Stakeholder views

Following the analysis framework we report the findings of stakeholder interviews as their views on the initial conditions of the project, what value they anticipated to gain from the project, the collaboration between the stakeholders (named value co-creation), the outcomes categorised as innovation, knowledge and relational outcomes, and how they summarised those as value created. Under the heading value captured whether and how outcomes were utilised by the stakeholders is described, and explanations to challenges observed is sought by identifying ownership of project outcomes and prospects for their continued utilisation. Finally, boundary conditions explaining successes and failures were identified.

#### Initial conditions

At the start of the project, there was a lively, largely positive, discussion on Value-Based Health Care, and a shared view that Sweden, with over 100 quality registries, had easy access to outcomes data and was well-positioned to be an early implementer of outcomes-oriented reimbursement systems. Interviewees saw an opportunity to promote improvement and develop new and meaningful forms of managing healthcare delivery based on trustworthy measurement. The possibility to involve many stakeholders in the project was a strength.

“…the ones who drove this forward believed very much in the idea of value-based healthcare, and that it would reform Swedish health care… …great advantage that we brought together people from different counties and from different competencies… …those who were good at research, analysis, data… …can we take a holistic approach to also have follow-up data via other systems?” (#6)

#### Anticipation of value

Interviewees had, by and large, high hopes for the project. With access to multiple data sources, it would be possible to combine and enable big data analysis. Follow-up of consolidated data would be done nationally, combining a national perspective with regional engagement. This would create the possibility to design Value-Based reimbursement or to change to an outcomes or quality-oriented health system governance – a novel way of holding providers accountable and promoting patient choice. It would offer participants an opportunity to better understand existing data, make use of new data, and seek ways to develop more cost-effective services, thus stimulating the improvement of Swedish healthcare.

“…so that you could really benchmark in a proper way with case-mix adjustments and, where you had the resources and where you saw potential to improve, and also be able to identify the hospitals or management that had good results and ask, what do you do to achieve these results? And stimulate work process improvement. So that was the ambition from our region, to allow and stimulate, in a good way, work improvement both on a regional level…” (#8)

#### Value co-creation

The *collaborative process* was promoted through an initial collective endorsement of the project aims and the stated research questions. Enabling choice of relevant indicators and comparisons was valued. The access to registry data and possibility to complement those with data from other sources were important success factors. Collaboration with relevant stakeholders, who represented all parts of the patient pathway, was viewed as fruitful as it brought in new and complementary perspectives and competencies, generated new ideas, and was a source of inspiration.

Expert groups were guided competently by the company to apply a systematic process to describe and analyse patient pathways in the eight patient groups. Relevant indicators were defined and sophisticated algorithms for case-mix standardisation were developed. This “SVEUS model” for managing outcomes and resource data was designed and continuously refined throughout the project period. The validity of data was scrutinised, and interpretations were thoroughly discussed. The high attention to methods and technical solutions was found especially valuable. Discussions on how to implement the insights and the tools in regional healthcare contexts were intense in several of the groups.

“We had agendas and we knew what we should reach, so it was extremely effective and creative and positive… …we were sort of going to get to relevant clinical variables and such. It was a very good structure all around… …I reported both to the XX region, and said region had groups too, those among us who were included in these different processes, the different diagnoses. We met regularly and exchanged experiences.” (#4)

#### Innovation outcomes

As to innovation outcomes, stakeholders referred to the project deliverables, listed in the Case description above.

#### Knowledge outcomes

The following knowledge outcomes were described: The “SVEUS model”, information on the legal requirements and restrictions regulating data acquisition and combination, insights on the importance of case-mix adjustments, and how benchmarking could promote improvement and outcomes-based governance.

“…differences between hospitals in outcomes were an extreme, for clinicians, eye-opener… … [the possibility to] based on specific characteristics define outcomes on a daily basis. Pedagogically this is fantastically useful in showing how healthcare varies.” (#4)

“It gave new insights… enthusiasm within the professions that, oh my god there is so much here which we can do, there is a large improvement potential that we did not even know existed, because we lack the dimensions in the available quality registries.” (#1)

#### Relational outcomes

Two major categories were derived from the interview data: collaboration and implementation.

Collaboration between specialists, seen as highly competent, and between different stakeholders along patient pathways functioned well. These collaborations were facilitated by a shared vision, the perceived relevance of case-mix adjustments for benchmarking, a well-structured process, the fact that every successful interaction made continued collaboration easier, and meaningful measurements.

“There were people from the “healthcare chain” with different competencies, as in my case being a researcher and knowledgeable in registries and variables. Then there were those with more clinical expertise, and from other areas too. It was well put together. There was good drive in it, in the work.” (#6)

Factors that impeded collaboration were expert groups with little diversity in terms of experience and competence, and difficulties to get all the regions and registries to work together and to involve local actors.

As to the company, it was highly appreciated for its competent staff and effective project management, although the fact that it is a private enterprise also generated some suspicion and distrust.

“But then in the third step of the project, the aim to make it a daily work reality, never occurred. We tried telling people about the project to everyone within the group, when it was on a clinical and hospital level. But that mostly only happened when you had a chance. Otherwise, all information went via the bigger meetings that were set up by IVBAR” (#4)

#### Value created

The project’s successful approach to linking registries and combining data from many sources increased the value of quality registries. Interviewees also described that it led to new insights on the improvement potential of data beyond quality registries, and on how to run improvement initiatives.

As an additional benefit, work in the SVEUS obstetrics group led to the establishment of a new registry on ruptures during delivery.

Legal and technical requirements for data acquisition and analysis were clarified, making it possible to produce benchmarking reports across the participating regions with increased validity thanks to sophisticated case-mix adjustments. This set the scene for real-time follow-up and visualisation of performance data that stimulated quality and process improvement.

SVEUS highlighted the usefulness of combining information on quality (outcomes) and costs, although the decision to downplay outcomes-based reimbursement diminished the value of the project.

The tangible deliverables of the project (the benchmarking reports covering eight patient groups and the automated analysis platform) were the most concrete expressions of value.

“…with the data we have, we have shown that it is possible to have an analysis, an automated analysis platform that examines health outcomes, quality and resource expenditure simultaneously.” (#7)

“when you could give feedback in this way, very quickly, it created a large commitment around improvement work on a clinical level.” (#9)

“…within health care you have always tried to blame your case-mix… there has not been any willingness to change within care because people do not really believe in the outcomes. And in this context having a possibility to gain more reliable outcome data, which we did from SVEUS… …that was motivational enough according to me.” (#5)

#### Value captured

Results of the project that were assessed as having an implementation potential with high sustainability potential were the case-mix adjustment algorithms and the analysis platform as well as its underlying conceptual model. Benchmarking reports were described as trustworthy. They showed that resources were not used optimally and confirmed suspected practice variations. Interviewees especially noted that highlighting complication rates led to fruitful discussions about those variations.

“… managed to get a real time follow up and continuous updating of data from many sources, where there is like an online feedback directly to our care providers and regions which supports operation development, follow-up, comparison…” (#7)

“Because they use the material that we created, the algorithms that were developed in the project are in that software. But there has not been any continuation regarding cooperation.” (#10)

However, regarding the implementation of deliverables, only three regions had used, and to varying degrees, the benchmarking information in practice. This was seen as a consequence of the project being carried out as research. Further spread within regions was hampered by poor communication and lack of senior management support. The company managed to raise international interest in the “SVEUS model” and the analysis platform, but not in Sweden.

“…Something that we failed to do with SVEUS and which I think had great consequences in the end, was the communication around the project under the second half.” (#9)

Another reason for reluctance among regions to move forward was that many awaited the Swedish Association of Local Authorities and Regions (SALAR) to integrate the project deliverables into its National Knowledge Management Centre, but that decision was delayed, and, in the end, never made.

“…It was then inferred that if you couldn´t get SVEUS into the national organization of knowledge, there was no use in moving forward with it. There were a few regions wanting to pursue while others clearly stated that they were not interested if SKL (Swedish Association of Local Authorities and Regions) did not adopt SVEUS. So basically, it failed because of SVEUS not being included in the SKL budget.” (#9)

#### Ownership

Despite the active engagement of stakeholders, questions regarding ownership, in particular outcomes data and deliverables, created confusion. Responsibilities among stakeholders were described as unclear. Questions were raised about who or which institutions owned the collected data. Participating regions saw themselves as future owners, although the systems were intended to be open source, a requirement by the Department of Health. Access to the analysis platform was experienced as restricted, even though staff of the participating regions had unlimited access.

“ But still, each respective county owned the data, but in this case, it also became rather unclear… a big part of the data came from quality registries.” (#4)

#### Continuation

The original implementation plan was to introduce the analysis platform into routine use by integrating it into existing administrative information systems in the respective regions. Five regions carried out the technical implementation, but only one actually started to use the platform. To give all regions access to the platform, a suggestion was made to engage SALAR to distribute the system, but in the end, SALAR did not accept that task. The reasons for this discontinuation of project deliverable use were described to be the following:

The project was carried out as a research project, which created an implementation challenge at practical and conceptual levels. Although the benchmarking reports were found useful, they did not lead to wide-spread local improvement initiatives. Such activities would have shown the benefit and possibly raised a wider interest in the analysis platform - this was described as a missed learning opportunity. Finally, the project was too bold and pioneering – “it was ahead of its time”.

Actions mentioned that could potentially have promoted implementation and continuation were: Highlighting the relevance of case-mix adjusted performance data to healthcare professionals; the newly established Knowledge Management Centre at SALAR choosing to host the analysis platform; and an earlier start of implementation efforts locally.

A general precondition for continuation described by the interviewees was broader political and financial support and more local senior management involvement.

“We need something that is up to date with 24-hour intervals, coordination with quality registries, patient administrative systems, that is the future, that is the cockpit. To have national follow-up, which is online is what everybody needs, what everybody would use, and that gives answers straight away, where resources is otherwise used without even knowing what to ask and if the answer is reliable.” (#1)

“…implementation into management, that is where more consideration should be given earlier… to identify early, that if we create a good product, how should it be managed and who is the owner of it. Moreover, how do we get it in both financially but also used by several stakeholders? To adopt the results from a research project into reality, that has been the major challenge.” (#8)

“ … a big challenge was to go from a research and development project to… implementation and management, there are many challenges related to that… …we have tried to give it a national residence so that it may live on…” (#7)

#### Boundary conditions

The boundary conditions viewed as most influential were the legal restrictions concerning availability of, access to, and management of data. Favourable conditions were that members in the expert groups knew each other from previous professional contacts, and the company managing the project was held in high regard. The “SVEUS model” was deemed important and, thus, the project work found to be meaningful, especially realizing the model in practice.

Less favourable conditions cited were the large number of stakeholders and their different levels of confidence in the ability to achieve the aims of the project, which interviews described as hampering progress. There was resistance among quality registries. There was some fear among stakeholders that benchmarking would lead to negative exposure. Reorganisations in regions and changes among key actors disrupted continuity.

The project was launched at a time when there was great general interest in VBHC. Soon afterwards, the entire concept, in particular the use of patient data, was heavily criticised in mass media, which changed the tune among healthcare professionals, too. VBHC also became connected in the minds of many to patient choice reforms and privatisation, which did not fit the agenda of the newly elected government in 2014.

“ …worried that the data would be handled in a wrongful manner. …simultaneously, this discussion regarding consult cooperation within various projects generally in Sweden. And this situation with YY hospital and what has been brought up in mass media. …the demand was incredible from both legal, economic, and other competence to see, can we at all take over such a common [project] with several regions?” (#7)

## Discussion

We summarise the findings of our analysis as follows: The multiple stakeholder innovation network described in this case was successful in reaching the intended *deliverables* in the form of eight benchmarking reports that applied sophisticated case-mix adjustment algorithms and an analysis platform for managing data and visualising information. As the deliverables were appreciated by all stakeholders and seen as potentially promoting improvement efforts in their organisations, these results represented the *value created* by the network. However, *value capture*, i.e. successful innovation implementation and spread of the tools produced, was *not* achieved.

It is striking that the stakeholders assessed the initial conditions, their anticipations of the project, and the collaboration between stakeholders during the project (value co-creation) in positive terms only. The views of the stakeholders were much more mixed when asked about implementation and spread. Clues about this shift in attitudes can be found under the theme “Relational outcomes”. Collaboration had been enhanced by a shared vision, collegial respect, and the perceived usefulness of the new tools that were developed. But participants noted the lack of interest among regions to actively implement the project deliverables, as the project was defined as research.

This is interesting given the fact that the project would, otherwise, not have been allowed to link the different databases, which was a prerequisite for the design of the case-mix adjustment algorithms. So, in a Catch-22, what made the most highly valued outcomes of the project possible, was also described as the greatest hinder to implementation and sustainability.

These observations are partly in line with the findings of Reypens et al.^9^ Value creation took place in the working groups characterised by efficient coordination by the consultancy company staff, a consultative approach, with high respect for member competencies and thus a willingness to compromise. But despite high initial anticipations of value and a positive assessment of the outcomes wider application did not materialise.

One might initially conclude that different mechanisms need to be applied to promote innovation generation and innovation implementation. This comes as no surprise, given the well-known challenge to get research results into practice.^16^ The original interest in VBHC and outcomes-related reimbursement led to the involvement of administrators and finance directors as regional representatives in the project steering group. As the focus changed from funding issues towards performance comparisons of outcomes to guide improvement, the interest of the regional representatives diminished. This, as shown in the case, diluted their sense of ownership. At the same time, attitudes towards VBHC turned negative, driven to a large extent by mass media.

This deteriorating interest among administrators was probably a crucial factor hindering implementation. The partly successful introduction of VBHC in a Swedish hospital required dedicated resources allocated to support the implementation, active measures that engage staff and dedicated leadership executed by senior managers.^17^ A similar implementation challenge was the introduction of telehealth in Canadian psychiatry, which was impeded by clinical challenges like barriers to information sharing and medicolegal concerns, technological and logistical challenges (incompatible information systems) and an insufficient readiness for change^18^ – remedies to those will all call for top management involvement.

In contrast to the administrators, the engagement of the health professionals in the expert groups, working with case-mix adjustment, and compiling the benchmarking reports, was preserved throughout the project. These professionals had their bases in clinical practice and were inspired by the now verified variations in outcomes that could be used to promote practice improvement. However, without access to the project tools, data driven discussions about what to improve and how to monitor impact became difficult. Those were important shortcomings, echoed by lessons from a “practical implementation” of VBHC in a Dutch hospital system, showing the importance of clinicians using outcomes as drivers of continuous quality improvement rather than point measures of clinical performance, the availability of tools promoting rapid cycles of process improvement and access to platforms for benchmarking.^19^ Those findings are in line with more general observations of implementation in clinical settings that emphasise the role of clear benefits to patients as the main motivator for clinicians to embrace change.^20^

Consequently, an alternative conclusion is to view this case not as a Catch-22, but more akin to the story of the Aristotelean hero with a tragic flaw (tension between views of research and practice), exacerbated by contextual forces (changing public sentiment regarding management trends). The way to address the tragic flaw would be if key stakeholders with a primary interest in applying an intended innovation are put in the driver’s seat, in this case the professionals involved in the expert groups. With that goal in mind, those professionals could have insisted that regional decision makers make the tools available to them. We have, in a study on a complex change process in a hospital setting, defined such an approach as a “professional path strategy” in that it resonates with the ethos of the professionals’ identity.^21^

Multiple stakeholders inevitably have different interests, ambitions, and anticipations regarding collaboration. These disparities can be overcome in a well-organised collaborative process, which was partly achieved in this case. But the danger is, as also became apparent, that uncertainty can develop regarding responsibilities and project ownership. The antidote would then be to recognise that, at least in the context of medicine, value capture is dependent on understanding different conceptualisations and motivations for value creation. If a key stakeholder with a major interest in value capture also has the expertise needed to generate the innovation, it might create more favourable conditions for the initiative to achieve its full potential. The role of other stakeholders would then be to support, provide resources, build infrastructures, and shield the process from undesired changes in boundary conditions.

This kind of a stakeholder constellation highlights some features of the collaborative processes in innovation networks found in other industries.^22–24^ One is the need for a systematic and well-organised process, paying attention to both deliverables and process outcomes.^22^ Another is the value of social interaction and collective learning.^23,24^ What this study adds to our understanding is that although “all animals are equal”, meaning that all stakeholders are important and contribute to collaboration, still “some animals are more equal than others”, as they hold the keys to successful implementation.
